# Dissecting the Space-Time Structure of Tree-Ring Datasets Using the Partial Triadic Analysis

**DOI:** 10.1371/journal.pone.0108332

**Published:** 2014-09-23

**Authors:** Jean-Pierre Rossi, Maxime Nardin, Martin Godefroid, Manuela Ruiz-Diaz, Anne-Sophie Sergent, Alejandro Martinez-Meier, Luc Pâques, Philippe Rozenberg

**Affiliations:** 1 Unité Mixte de Recherche 1062 Centre de Biologie pour la Gestion des Populations, Institut National Recherche Agronomique, Centre de Montpellier, France; 2 Unité de Recherche 588 Amélioration Génétique et Physiologie Forestières, Institut National Recherche Agronomique, Centre d’Orléans, France; 3 Parque Tecnológico Misiones, Universidad Nacional de Misiones, Misiones, Argentina; 4 Consejo Nacional de Investigaciones Científicas y Técnicas, Bariloche, Argentina; 5 Grupo de Ecologia Forestal, Instituto Nacional de Tecnología Agropecuaria, Estación Experimental Bariloche (INTA EEA Bariloche), Bariloche, Argentina; CNRS - Université Lyon 1, France

## Abstract

Tree-ring datasets are used in a variety of circumstances, including archeology, climatology, forest ecology, and wood technology. These data are based on microdensity profiles and consist of a set of tree-ring descriptors, such as ring width or early/latewood density, measured for a set of individual trees. Because successive rings correspond to successive years, the resulting dataset is a *ring variables* × *trees* × *time* datacube. Multivariate statistical analyses, such as principal component analysis, have been widely used for extracting worthwhile information from ring datasets, but they typically address two-way matrices, such as *ring variables* × *trees* or *ring variables* × *time*. Here, we explore the potential of the partial triadic analysis (PTA), a multivariate method dedicated to the analysis of three-way datasets, to apprehend the space-time structure of tree-ring datasets. We analyzed a set of 11 tree-ring descriptors measured in 149 georeferenced individuals of European larch (*Larix decidua* Miller) during the period of 1967–2007. The processing of densitometry profiles led to a set of ring descriptors for each tree and for each year from 1967–2007. The resulting three-way data table was subjected to two distinct analyses in order to explore i) the temporal evolution of spatial structures and ii) the spatial structure of temporal dynamics. We report the presence of a spatial structure common to the different years, highlighting the inter-individual variability of the ring descriptors at the stand scale. We found a temporal trajectory common to the trees that could be separated into a high and low frequency signal, corresponding to inter-annual variations possibly related to defoliation events and a long-term trend possibly related to climate change. We conclude that PTA is a powerful tool to unravel and hierarchize the different sources of variation within tree-ring datasets.

## Introduction

Tree-ring datasets are widely used to reconstruct histories of disturbance events and forest dynamics [1]–[3], infer large-scale patterns of climate variation (dendrochronology) [4]–[8], assess trends in tree growth and forest management options [9]–[11], and regulate wood production and wood quality by controlling site, silviculture, and genetics. Tree-ring data based on microdensity profiles are collected in stems of a set of individual trees, which contains a number of successive annual rings [12] related to the age of the tree, since a new ring is added each year. The most evident structure in a temperate tree ring, especially in conifers, is the earlywood-latewood succession. The light-colored, low-density earlywood is the first part of the ring, formed at the beginning of the growing season (spring and early summer), when temperature is mild, soil water content is high, and the photoperiod is increasing. The darker, higher-density latewood forms during the second part of the growing season (summer and early autumn), when temperature is higher, soil water content is lower, and the photoperiod is decreasing. Earlywood and latewood width and density are variable, and transition from earlywood to latewood is more or less gradual, affected by species, genetics, tree age, and environment, including climatic variation from the first part to the second part of the growing season. Ring width, earlywood width, latewood width, earlywood density, and latewood density are frequently used to describe a single ring [13]. A basic microdensity table for a single annual ring is a two-way matrix containing as many lines as the number of trees under study and as many columns as the number of variables used to describe each annual ring.

A tree-ring dataset is a three-way dataset of the form *ring variables* × *trees* × *time*. These datasets are often considered two-way matrices, such as *ring variables* × *trees* or *ring variables* × *time*, and subsequently analyzed by multivariate analyses such as principal component analysis (PCA) [14]. Unfortunately, this strategy provides only an incomplete picture of the multivariate space-time variation within the aforementioned datacube [15]. The recent decades have experienced the development of various tools to explore and interpret three- or higher way structure of the data. Popular multiway methods include models from the PARAFAC [16] and Tucker [17] families and alternative models such as the family of STATIS methods [18], [19]. The STATIS methods were introduced by L’Hermier des Plantes [20] and Robert and Escoufier [21] and developed by various authors [22], [23]. Partial triadic analysis is one of the simplest STATIS methods. It is derived from the triadic analysis [17] and was introduced in ecology under the name of “triadic analysis” by Thioulouse and Chessel [15]. Kroonenberg [24] further renamed the method “partial triadic analysis”, emphasizing the difference between the original triadic analysis. The PTA is an exploratory multivariable technique based on PCA. In the case of a spatial structure with repeated measurements, it allows for depicting of temporal variability of the multivariable spatial structure and/or the spatial structure of the temporal trajectories. The PTA has been used in a vast array of situations and disciplines including limnology [25]–[28], marine ecology [29], soil ecology [30]–[34], landscape ecology [35], hydrology [36], and food science [37], among others. To our knowledge, however, the potential of PTA has never been assessed in the framework of tree-ring data analysis.

The objective of this study was to show how this technique could be used to explore the variability in a forest tree stand (spatial structure), where each tree is described using several annual tree rings (repeated measurements). This dataset was derived from a set of 149 neighboring, georeferenced trees, constituting a small forest stand. One increment core was collected from each tree. A 41-year, annual-ring time series was obtained from each increment core. Each ring microdensity profile was described by means of 11 ring variables. Although not involved in PTA calculations per se, a large climate dataset was available [38] and could be used to explore possible correlations between average annual minimum and maximum temperatures (1967–2007) and some PTA outputs. We explored our data from two points of view: the analysis depicting the temporal evolution of spatial structures and that portraying the spatial structure of temporal dynamics.

## Statistical Background

**Figure 1:**
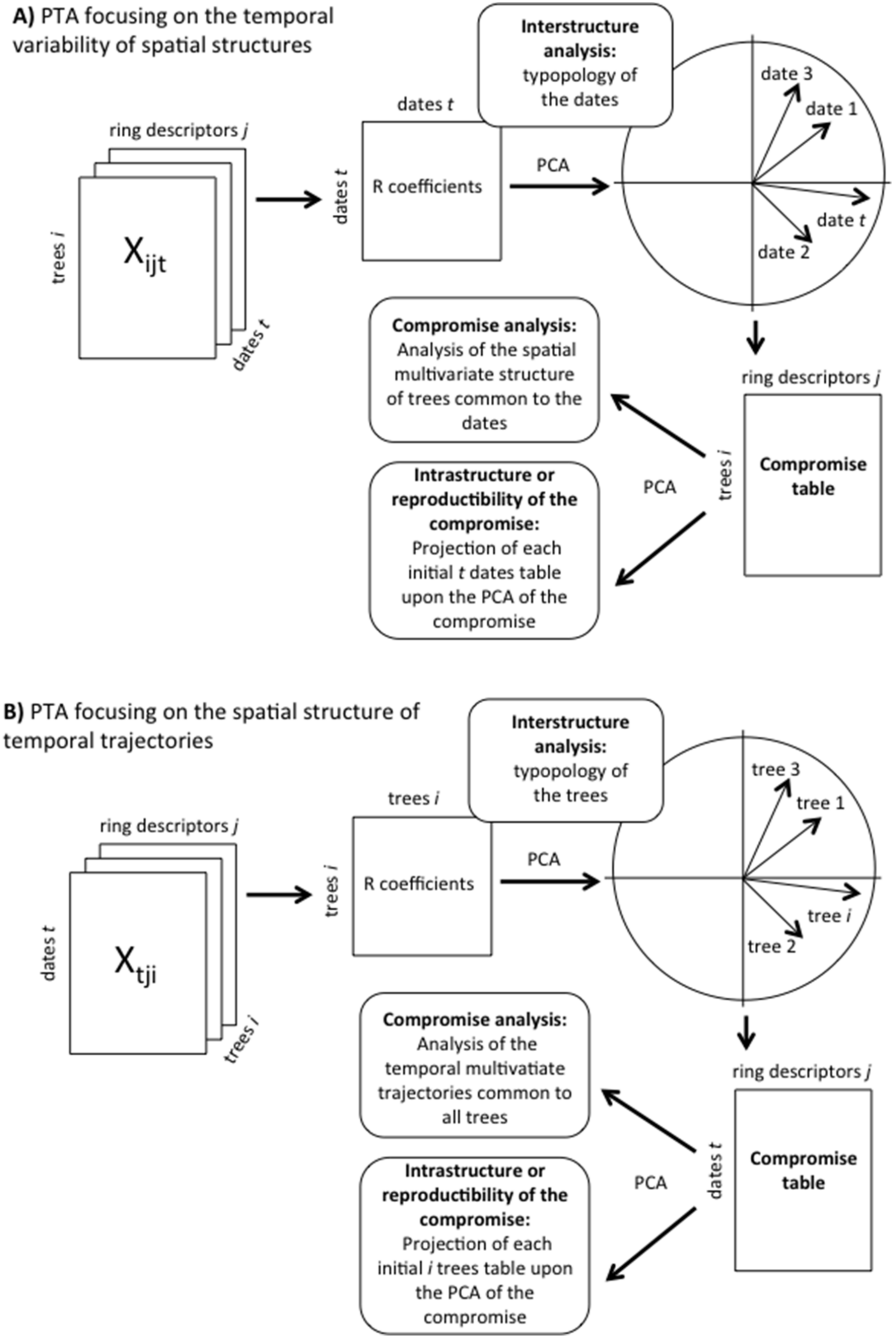
The partial triadic analysis is designed to analyze the realizations of a set of random variables (ring descriptors) measured on a set of points (trees) at different sampling occasions (dates). This corresponds to a three-way table with three subscripts (X_ijt_) standing for trees, descriptors, and dates, respectively. A given dataset can be analyzed from two complementary viewpoints: seeking for either the temporal evolution of spatial structures (1A) or the spatial structure of temporal dynamics (1B).

The theoretical background of PTA is available in various publications [15], [24], [25], [28], and readers are referred to these publications for a formal presentation of the method. We will focus here on an application in the context of tree-ring data analysis and will solely provide an overview of the statistical background. The PTA is designed to analyze the realizations of a set of random variables measured in the same individuals (trees in this case) at different sampling occasions (years of formation of the annual rings). It is based on PCA [14], [39] and processes a three-way table consisting of a data matrix with three subscripts (X_ijt_) that stand for trees, descriptors, and dates (Figure 1). The PTA searches for structures that are stable across a set of two-way tables derived from X_ijt_. This can be considered in two ways: either the focus is the *trees* × *ring descriptors* or the *dates* × *ring descriptors* tables (Figure 1). The first strategy highlights the temporal variability of ring microdensity profile spatial structures (Figure 1A), while the second indicates the spatial structure of temporal trajectories (Figure 1B). This paper reports both of these complementary points of view.

The PTA involves three steps: the interstructure, compromise, and intrastructure analyses [15], [23]. Readers are referred to previous work [23] for a formal definition of these terms. The goal of the interstructure is to make a typology of the tables. If we consider the *trees* × *descriptors* two-way tables, the interstructure yields a typology of the dates (Figure 1A). In that case, the typology is based on the analysis of the *trees* × *ring descriptors* tables taken as the individuals of PCA [23]. Data preprocessing is an important step that should be considered carefully [40]. The two-way tables X (either *trees* × *ring descriptors* or *dates* × *ring descriptors*) were centered and scaled in order to remove the differences among ring descriptors due to different measurement units or scales without altering the differences between trees or sampling dates. The mean of the correlation coefficients for similar variable *j* between X_k_ and X_l_ defines the vectorial correlation coefficient R between these tables. The R coefficient ranges from –1 to +1, since it is the mean of a set of correlation coefficients. The date typology is obtained from the non-centered PCA of the *t* × *t* matrix of the inter-date R coefficients [23] (Figure 1A).

The second step of the PTA consists of analyzing the compromise table, which is derived from the positive eigenvectors of the PCA of the interstructure (Figure 1). It contains the factorial coordinates of the trees (dates) for each microdensity profile descriptor (see [25] for a graphical representation). The compromise table is a two-way table summarizing the initial three-way datacube and is analyzed by means of PCA to depict the multivariate structure common to all tables. If we focus on the temporal variability of the *trees* × *ring descriptors* two-way table, the compromise table will consist of a *trees* × *ring descriptors* two-way table. In this example, it will encapsulate the multivariable spatial structure common to dates (Figure 1A and Figure 1 in [30]).

The last step of PTA is called the intrastructure [15]. It consists of projecting the initial two-way matrices as complementary tables upon the axis of the PCA of the compromise. This allows assessing which table fits (or does not fit) the structure encapsulated in the compromise. Again, if we consider analyzing the trees × *ring descriptors* two-way tables along dates, the intrastructure provides a picture of the departure of the spatial structure observed at each date from the spatial structure common to all sampling occasions (Figure 1).

## Materials and Methods

### Ethics statements

This study was approved by the National des Forêts and allowed by the municipality of Villar-Saint-Pancrace (Hautes-Alpes, France). This survey did not involve endangered or protected species.

### Site and species

The study site is located close to Villar-Saint-Pancrace (44°52′N, 6°41′E; Hautes-Alpes, France) in the French Alps. The sampling site used in this study is one of four plots of an altitudinal gradient extending from 1350–2300 m above sea level (asl). This experimental site was sampled from 2008–2012 with the goal of studying the adaptation of larch to climate. The altitude of the sloping survey plot ranges from 1640–1683 m, and the vegetation is a continuous natural population of European larch (*Larix decidua* Miller). Increment cores were collected at breast height from 149 trees and used then to study spatial and temporal relationships between annual ring characteristics and climate. Genetic markers were also used to investigate genetic diversity and local adaptations. We focused on the period of 1967–2007, during which the total annual precipitation ranged from 425.5–1078.2 mm in Briançon (44°53′N, 6°38′E), where the nearest “Météo-France” climate station is located. The mean annual temperature, mean annual temperature of the coldest month, mean annual temperature of the warmest month, and mean number of frosty days were 6.32°C, –1.49°C, 15.37°C, and 170, respectively, during the 1967–2007 period. The soil is a colluviosol type. More details are available in [38].

### Sampling and measurements

The area of the plot was 5815 m^2^ and featured 149 trees homogeneously distributed in space with an inter-individual separating distance ranging from 1.1–112.4 m. The stand density was 354 trees/ha, the mean tree age was approximately 150 years, and the average tree height was about 27 m. Increment cores collected at breast height (see details below) provided annual ring data for each tree from 1967–2007 (i.e., 41 successive years [38]).

The numbers and characteristics of the annual rings were estimated using a microdensitometry approach, which also allowed estimating the age of each tree. Pith-to-bark radial increment cores were collected at breast height using a 5.5-mm Pressler increment borer following a constant north-south orientation. The samples were dried to 12% water content and X-rayed [41], [42]. The X-ray films were scanned at 4000 dpi. The microdensity profiles were obtained using the software WINDENDRO (Windendro 2008e, Regent Instruments Canada, Inc.) [42]. The microdensity profiles were cross-dated (Interdat.exe version 1.1, Dupouey J-L, unpublished work), and the number of rings in each increment core was counted. The ring variables (Table 1) were measured following methods described previously [43]. Most variables are conventional ones, but others rarely employed (i.e., the three standard deviation variables) were used. The majority of these descriptors are based on the earlywood-latewood model, which divides the ring into two successive and contrasting parts [44].

**Table 1:** Ring variables used in the study.

Abbreviation	Definition	Unit
RW	Ring width: the length (or width) of the annual ring along the radius	mm
LW	Latewood percentage: the length of the latewood part of the annual ring alongthe radius divided by the ring width (RW). Earlywood percentage is 1 - LW.	mm
RD	Mean of the ring microdensity profile	g/dm^3^
ED	Mean of the earlywood part of the ring microdensity profile	g/dm^3^
LD	Mean of the latewood part of the ring microdensity profile	g/dm^3^
MID	Minimum ring density	g/dm^3^
MAD	Maximum ring density	g/dm^3^
Co	Density contrast: maximum ring density minus minimum ring density	g/dm^3^
RSD	Standard deviation of the ring microdensity profile	g/dm^3^
ESD	Standard deviation of the earlywood part of the ring microdensity profile	g/dm^3^
LSD	Standard deviation of the latewood part of the ring microdensity profile	g/dm^3^

The ring variables describe some aspects of the structure of the whole annual ring. The earlywood variables describe the structure of the wood formed during the first part of the growing season when the temperature is mild, the soil water content is high, and the photoperiod is increasing. The latewood variables describe the structure of the second part of the ring, which is formed during the second part of the growing season when the temperature is higher, the soil water content is lower, and the photoperiod is decreasing. The emphasis given to ring variables differs according to the discipline and the objective of the study. For example, earlywood is known to be by far the most conductive part of the ring; thus, earlywood density is important for sap conduction. Latewood maximum density is related to summer temperature and is used by dendrochronologists to reconstruct past climates. Latewood proportion and latewood density have been found to be strongly related to wood mechanical properties, such as the modulus of elasticity, and can be used to estimate wood value.

The ring data used in this study are provided as supporting information (Dataset S1) and their change from 1967 to 2007 is shown in Figure S1.

### Data analysis

The ontological tree-ring age has potentially strong effects on tree-ring width and wood properties [45]. We explored this effect with our time series running from 1967–2007. In these series, tree age ranged from approximately 100–250 yr. As a consequence, the annual ring time series covered a 41-yr period corresponding to cambial ages ranging from approximately 50–250 yr. Because the effect of cambial age upon ring variables is significant for ages <30 yr, we did not observe such an effect in our dataset [38].

All data analyses and graphics preparations were performed using the R statistical software package [46]. The PTA was performed using the R package ade4 [47].

The score of the trees upon the first axis of the PCA of the compromise table was analyzed by means of the Moran’s *I* autocorrelogram. We assessed the presence of a significant spatial structure i.e. a significant departure from randomness, using 1000 random permutations following [48]. The global significance of the correlogram was statistically assessed using Holm’s correction for multiple testing as described in Legendre and Legendre [14]. Correlograms were computed using the R package ncf [49].

## Results

### Depicting the temporal evolution of spatial structures

The first PTA described here aimed at depicting the temporal evolution of spatial structures (Figure 1A). In that case, the interstructure analysis allowed for weighing the dates, thus providing a compromise matrix where more weight is given to dates exhibiting similar spatial structures. As a consequence, the compromise picks up spatial structures (i.e., a spatial typology common to the sampling dates) (Figure 1A). The intrastructure of this PTA will assess the reproducibility of the former compromise, which attempts to assess which dates fit the global spatial structure summarized in the compromise (model) (and which do not) and to identify the ring variables that might explain these patterns (Figure 1A).

#### Interstructure

**Figure 2:**
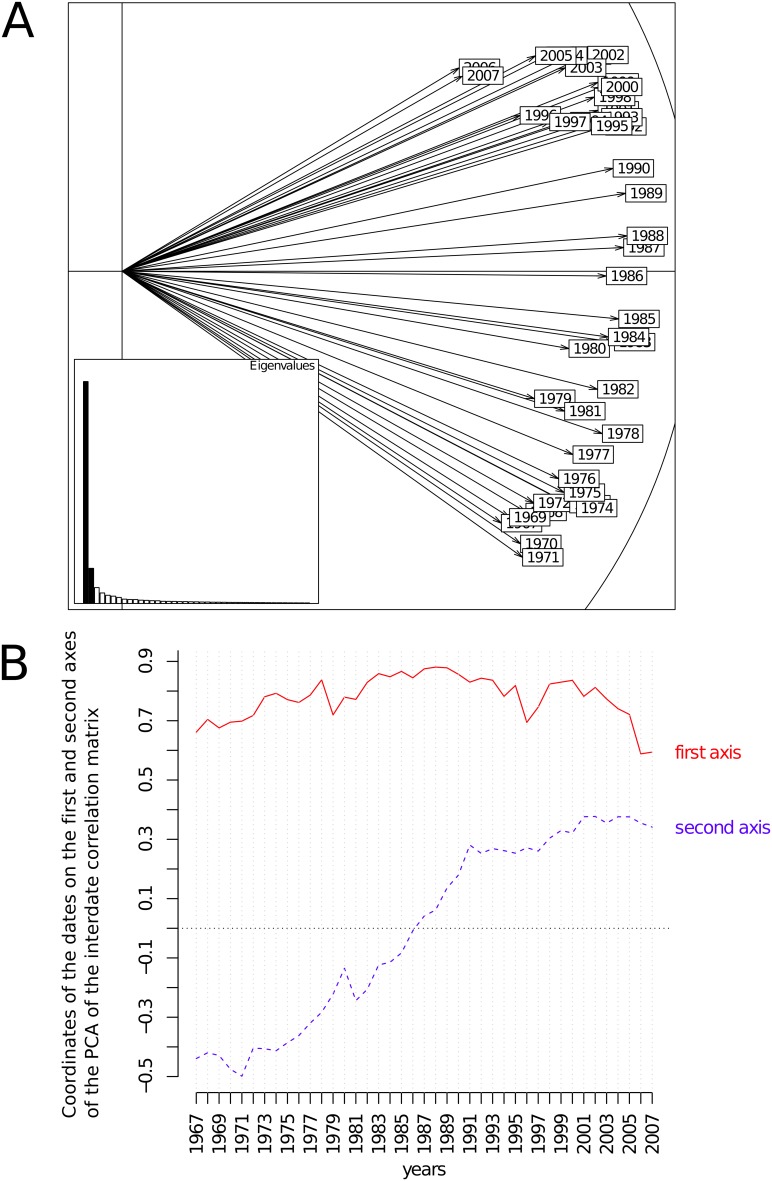
Interstructure analysis of the partial triadic analysis depicting the temporal evolution of spatial structures. A. Scores of the sampling dates upon the principal components of the principal component analysis of the inter-date correlation matrix. The first principal component (horizontal axis) represented 61.2% of the inertia. The second component (vertical axis) accounted for 9.7% of the total inertia. B. Scores of the sampling dates upon both first and second axes as a function of the years.

The interstructure table was a 41×41 square matrix containing the vectorial correlation (R) between the *trees* × ring *descriptors* sub-matrices (Figure 1A). The PCA of this matrix yielded a clear typology, with the first two principal components accounting for 61.2 and 9.7% of the total inertia, respectively. The corresponding correlation circle is given in Figure 2A. All the dates displayed positive scores upon axis 1, indicating the presence of a structure common to all dates. The case of the second axis was somewhat different, as dates displayed positive and negative values on this axis. Figure 2B shows how coordinates of dates upon both axes changed from 1967–2007. Whereas no clear trend was evident from axis 1, overall, the dates displayed increasing coordinates upon the second axis over the annual ring time series.

#### Compromise

**Figure 3:**
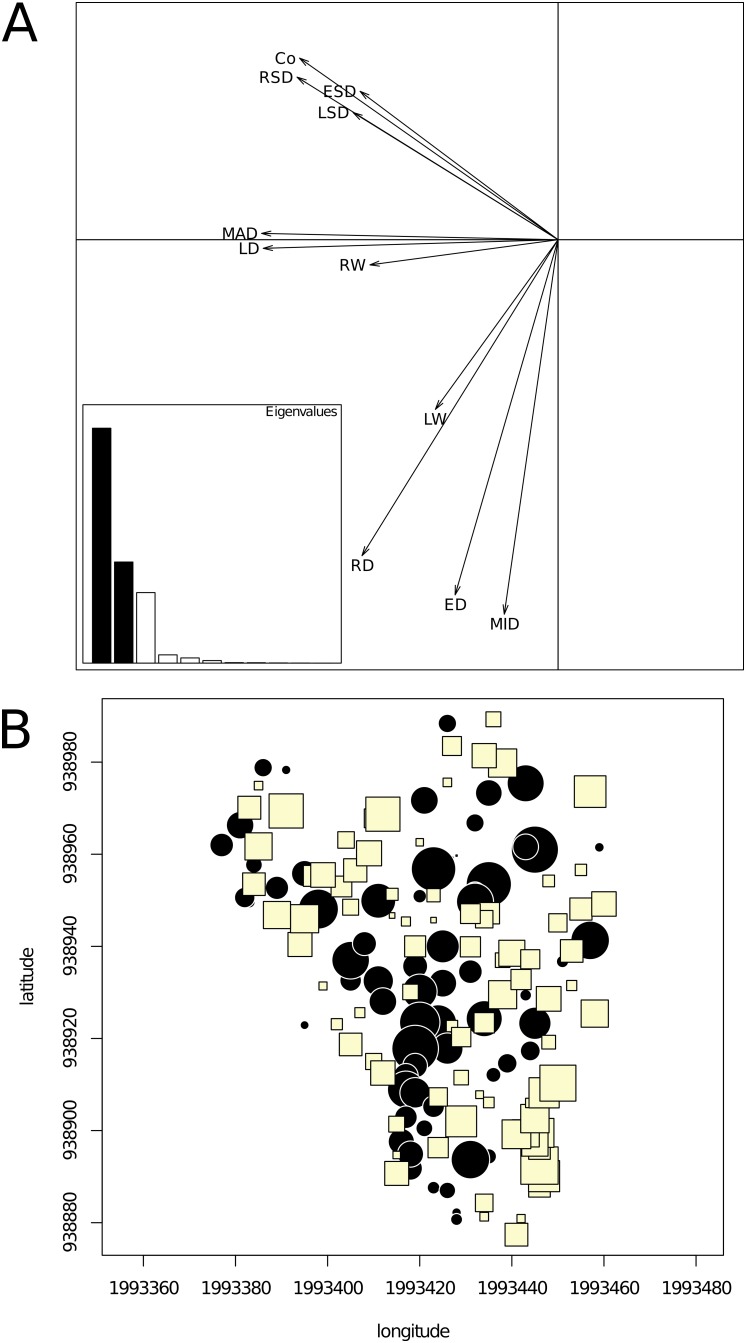
Compromise analysis of the partial triadic analysis depicting the temporal evolution of spatial structures. A. Correlation circle of the principal component analysis of the compromise table (a *tree* × *ring* descriptors table). The first (horizontal) and second (vertical) principal components accounted for 55.4 and 23.9% of the inertia, respectively. B. Map of the tree score upon the first axis of the PCA of the compromise table showing a strong spatial structure with alternating humps and bumps corresponding to patches of negative and positive scores. The symbol size is proportional to the absolute value of the score. Circles (squares) stand for positive (negative) values.

The compromise table associated with the first component of the PCA of the interstructure table was a *ring variables* × *tree* two-way table. The correlation circle of the PCA of this compromise is shown in Figure 3A. The first two principal components accounted for 55.4 and 23.9% of the total inertia, respectively. The first axis showed opposite effects between trees having higher latewood density (LD) and maximum ring density (MAD) with trees for which these variables exhibited lower values. These variables were associated with descriptors of intra-ring variation, including the standard deviation of ring microdensity profile (RSD) and density contrast (Co), and, to a lesser extent, the standard deviations of the latewood and earlywood parts of the ring microdensity profiles (LSD and ESD, respectively), which conveyed larger variability in the latewood density compared to earlywood. The second axis separated trees with higher intra-ring density variations (RSD, Co, LSD, and ESD) from trees with higher earlywood density (ED), higher minimum ring density (MID), and, to a lesser extent, higher mean ring density (RD).

Since the compromise encapsulates spatial information common to all annual rings and we are dealing with georeferenced sampling points, we could explicitly test for departure from spatial randomness. We first employed a graphical approach by mapping tree score upon the first axis of the PCA in the geographical space (Figure 3B). The values appeared to be strongly spatially correlated with trees, forming patches of positive values alternating with gaps (negative values). The presence of spatial autocorrelation was assessed by means of Moran’s *I* correlogram (Figure S2), which revealed highly significant departure from randomness (*p*<0.05 after Holm’s correction). The spatial correlation analysis provided an interesting clue toward the spatial scale of these structures that ranged below roughly 30–40 m, as indicated by the shape of Moran’s *I* correlogram. This means that the spatial structure isolated by PTA, which was common to all sampling occasions, corresponded to patches of trees with higher values of MAD, LD, RW, Co, and RSD (i.e., negative coordinates upon axis 1) and gaps of trees with lower values for these ring descriptors (i.e., positive coordinates upon axis 1).

#### Intrastructure

**Figure 4:**
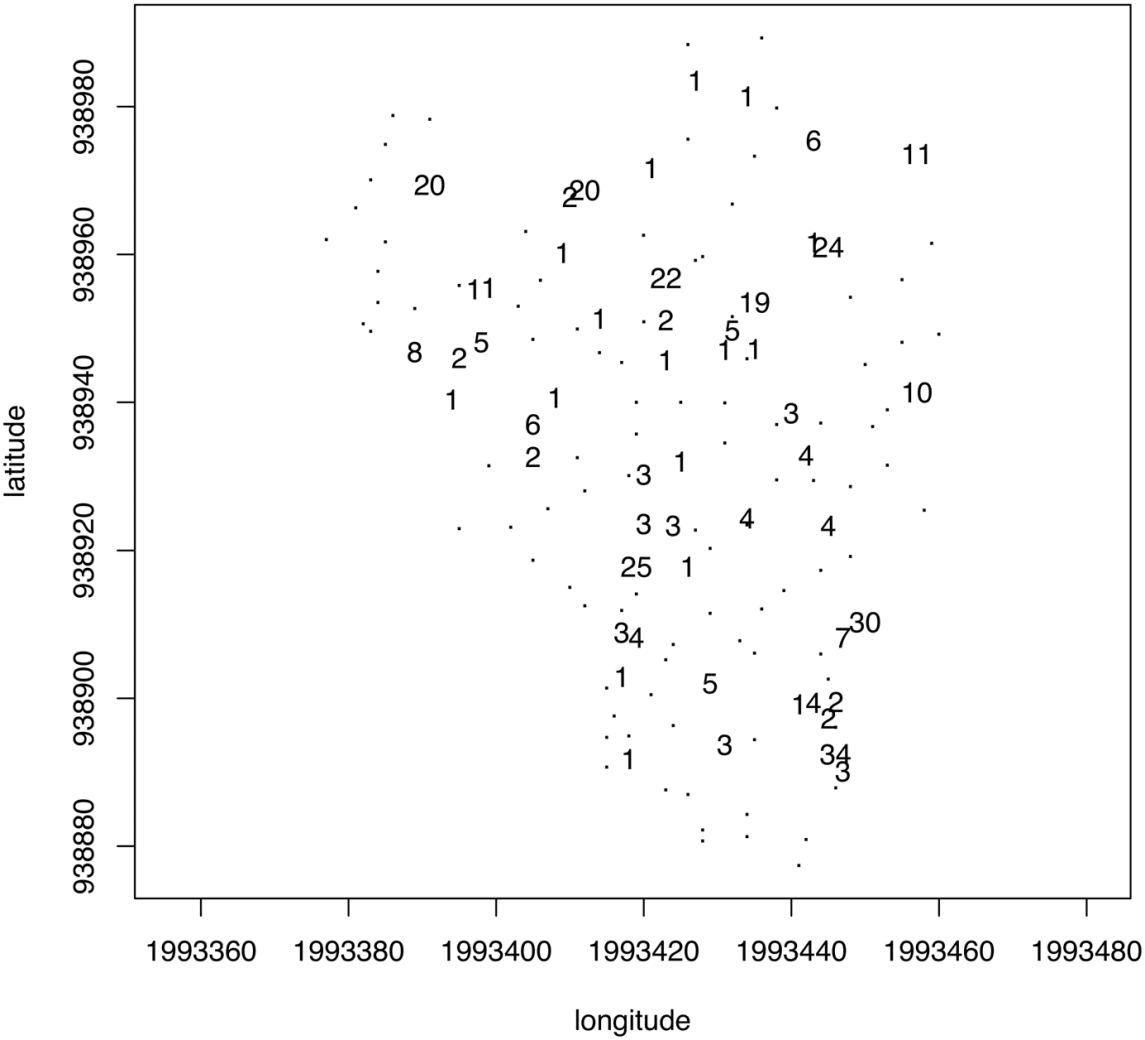
Intrastructure analysis of the partial triadic analysis depicting the temporal evolution of spatial structures. Map of the trees showing the number of times each one fell outside of the 95% envelopes of the tree coordinates projected onto the first axis of the principal component analysis of the compromise for each date.

**Figure 5:**
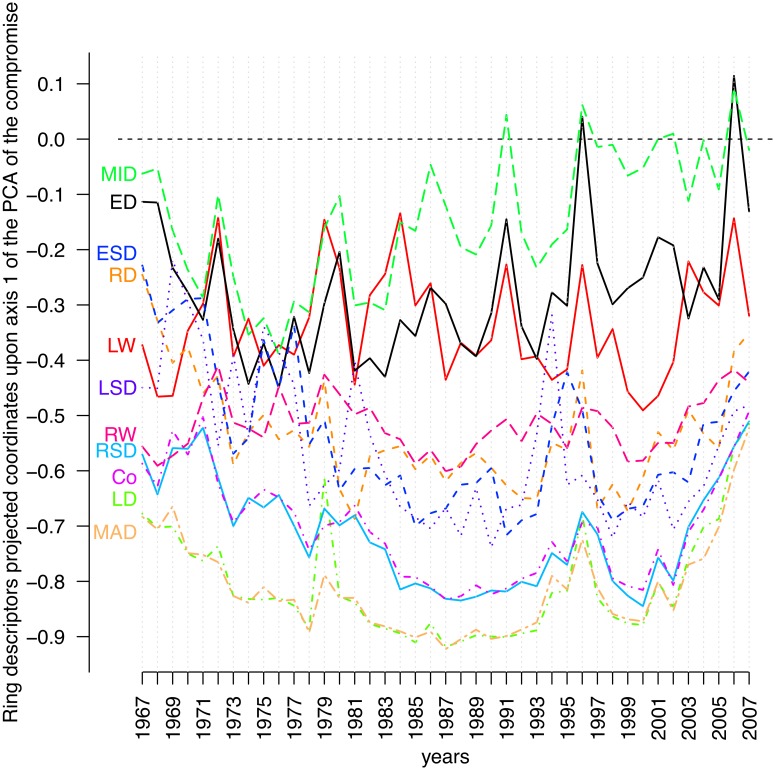
Intrastructure analysis of the partial triadic analysis depicting the temporal evolution of spatial structures. Scatter plot showing the coordinates of the ring variables projected onto the first axis of the principal component analysis of the compromise table across dates.

The last step of PTA is intended to reveal which original table exhibits departures from (or fits) the model expressed through the compromise. The original tables consisting of 41 *trees* × *ring* descriptor tables were projected as complementary tables onto the first axis of the PCA of the first compromise table. At each sampling date, we computed the quantiles (for probabilities of 0.025 and 0.975) of the coordinates of the 149 trees projected upon the first axis of the PCA of the compromise. These values were used as bounds to identify the trees that exhibited the largest differences between a model common to all dates (i.e., the compromise) (Figure 4). Fifty-three trees fell out of that envelope at least on one date, 18 of which fell out only once. A small number of trees differed from the model for the majority of the sampling dates (e.g., 34 and 30 values out of the envelope). Nonetheless, the map shown in Figure 4 revealed that the distribution of these trees displayed no particular spatial pattern at the plot scale. Figure 5 shows the coordinates of each ring descriptor as projected upon the first axis of the PCA of the compromise table for each date. These trajectories fluctuated according to dates, but their position with respect to each other did not change much and followed the pattern displayed in the correlation circle of the PCA of the compromise (Figure 3A).

### Depicting the spatial structure of temporal dynamics (trajectories)

The second set of analyses focused on the construction of a temporal typology common to each tree, which is equivalent to extracting the spatially stable part of the temporal structure. For that purpose, we considered the initial tables of *dates* × *ring* descriptors and performed the PTA on the interstructure table containing inter-tree R coefficients (Figure 1B).

#### Interstructure

The interstructure table was a 149×149 square matrix containing the vectorial correlation R between the *dates* × *ring* descriptor sub-matrices (Figure 1B). The PCA of the interstructure matrix led to the correlation circle (not shown), where the two first principal components accounted for 35.0 and 7.0% of the total inertia, respectively. The correlation circle showed that all trees exhibited a common temporal dynamic, although it was expressed with more or less intensity depending on the tree considered.

#### Compromise

**Figure 6:**
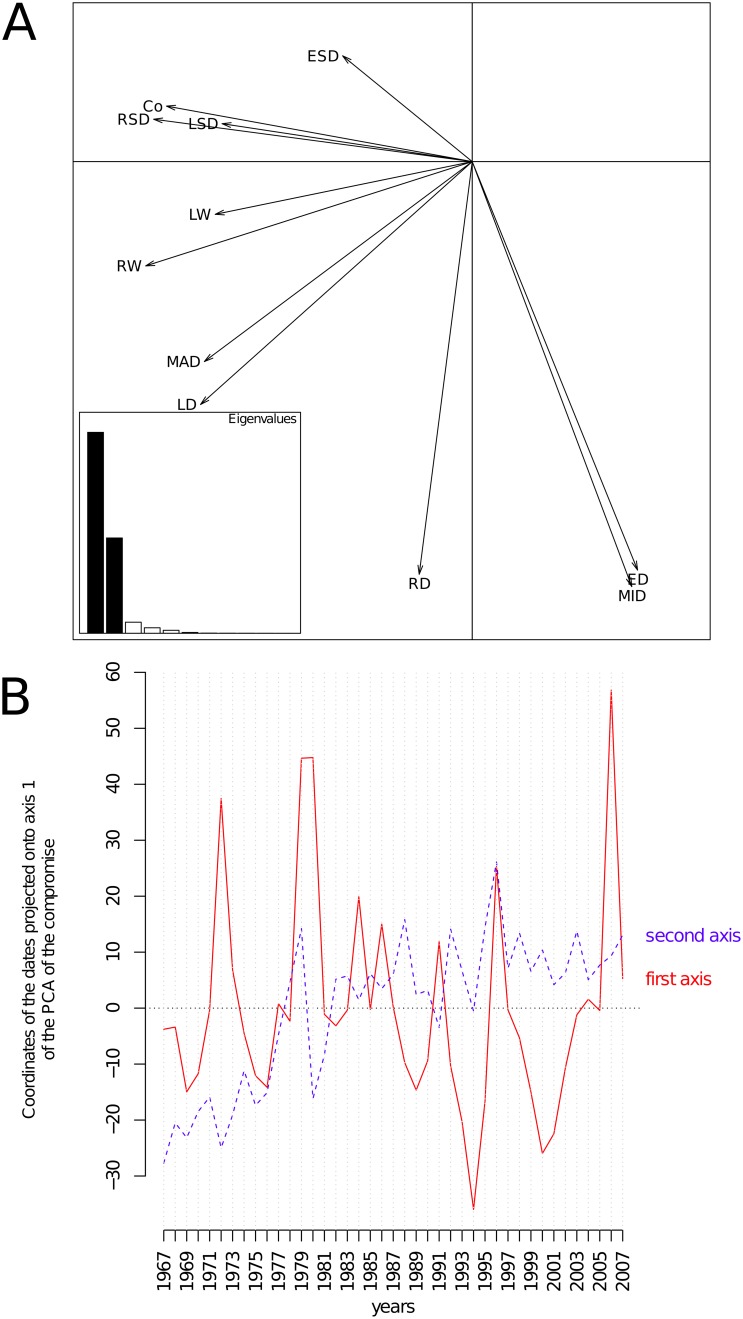
Compromise analysis of the partial triadic analysis depicting the spatial structure of temporal dynamics. Correlation circle of the principal component analysis (PCA) of the compromise table (a *date* × *descriptor* two-way table). The first principal component (horizontal axis) represented 63.4% of the inertia. The second component (vertical axis) accounted for 30.0% of the total inertia. The first axis expresses the opposition between dates where ring growth and latewood production was high with less favorable periods. B. Changes of dates coordinate upon the first (solid line) and second (dashed line) axes of the PCA of the compromise table. The coordinates of the dates onto the first axis showed no temporal trend but, rather, the presence of punctual events that strongly affected ring descriptors. Dates coordinated onto the second axis revealed a temporal trend over the period of the survey.

The compromise table associated with the first component of the previous interstructure analysis was a *date* × *ring descriptor* table, and the correlation circle of its PCA is shown in Figure 6. The first (horizontal) and second (vertical) components accounted for 63.4 and 30.0% of the total inertia, respectively. This correlation circle is very close to the one displayed in Figure 3A and expresses the same global pattern. The temporal typology common to all trees mainly corresponds to years where trees had higher values of RW, MAD, Co, RSD, and LD, as indicated by the first axis (Figure 6). Another source of variation common to all trees opposed years with high and low values of MID, ED, and RD (axis 2, Figure 6). A convenient way of displaying the patterns of the years with respect to the axis of the PCA of the compromise table consists of plotting the coordinates of each date upon both first and second axes as functions of the years (Figure 6B). From this plot, it can be seen that the first axis showed no clear temporal trend; a zigzag shape reflecting the alternating periods of high or low ring width, ring density, and latewood density is evident. Years 1972, 1979, 1980, 1996, and 2006 corresponded to high coordinates upon the first axis of the PCA of the compromise, which indicated particularly low growth and low latewood density. On the contrary, 1994 and 2000 were associated with better growth conditions. The coordinates of dates upon axis 2 smoothly increased from 1967–2007 in the form of a trend. There was a clear relationship with the mean daily minimum (r = 0.48, p = 0.0013) and maximum temperatures (r = 0.54, p = 0.0002) averaged over the growing season (March–September), as shown in Figure S3.

#### Intrastructure

**Figure 7:**
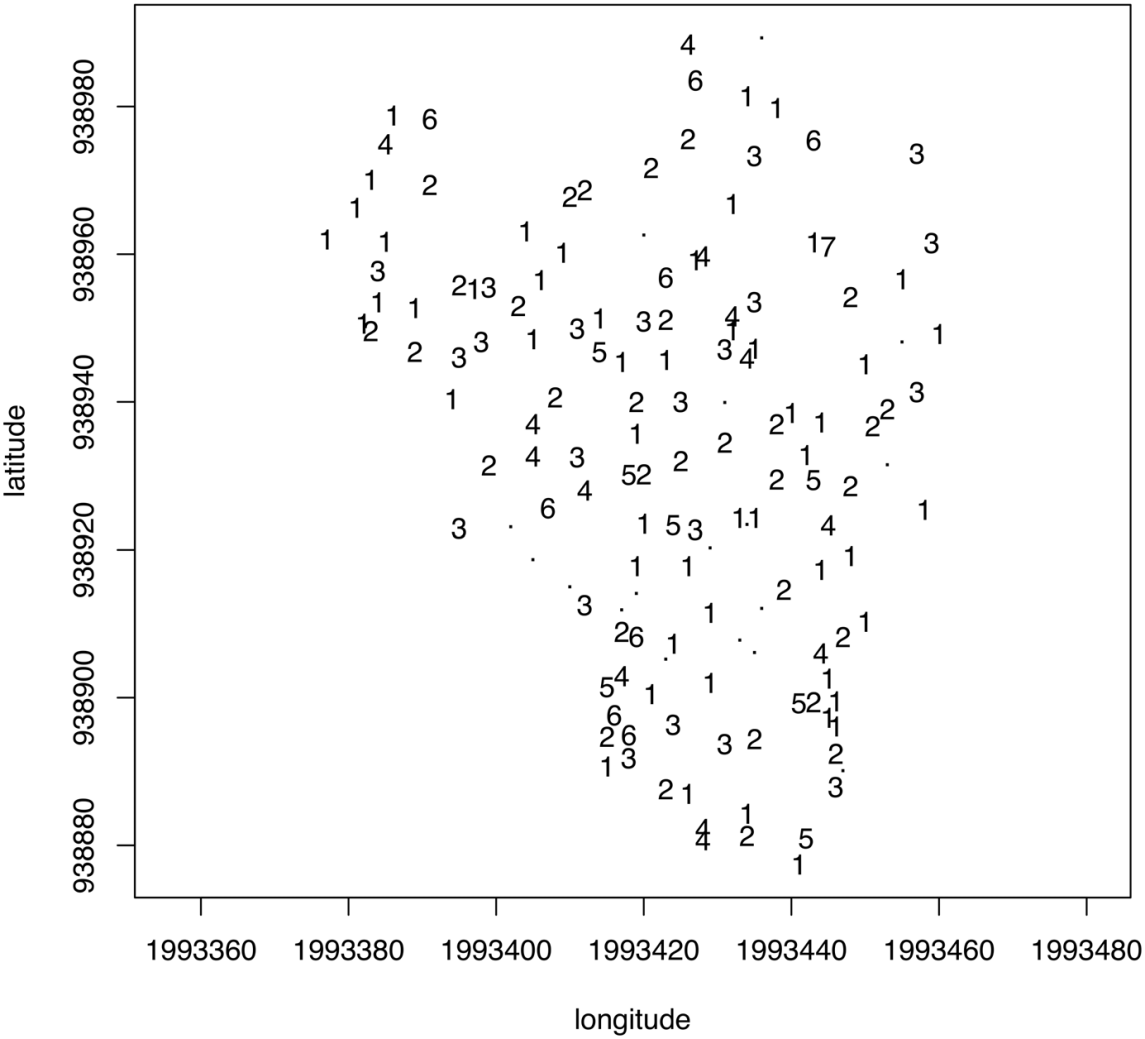
Intrastructure analysis of the partial triadic analysis depicting the spatial structure of temporal dynamics. Map of the trees showing the number of times each one fell outside the 95% envelopes of the tree coordinates projected onto the first axis of the principal component analysis of the compromise for each date.

The intrastructure of the PTA depicting the spatial structure of temporal dynamics indicates the departure of certain trees from the common model and the ring descriptors that could explain the phenomenon. The 149 original *dates* × *ring descriptor* data tables were projected upon the first axis of the PCA of the compromise. At each sampling date, we computed the quantiles (for probabilities of 0.025 and 0.975) of the coordinates of the 149 trees projected upon the first axis of the PCA of the compromise. These values were used as bounds to identify the trees that exhibited the largest differences between the model common to all trees (i.e., the compromise). A total of 132 trees (88.6%) fell outside the envelope at least once during the study period. Figure 7 shows the number of occasions that each tree fell outside the envelope. No spatial structure appears, which means that departure from the compromise is not spatially dependent.

Figure S4 shows the coordinates of each ring descriptor projected onto the first axis of the PCA of the compromise at each tree. For most of the ring descriptors, the coordinates upon axis 1 are fairly homogeneous, indicating a lack of spatial variability and limited departure from the structure encapsulated in the compromise. Some limited divergence with respect to the compromise appeared for the variable ED (negative values for some trees scattered across the plot), whereas ED had a positive coordinate onto the first axis of the PCA of the compromise (Figure 6A). Similarly, several trees displayed positive coordinates for ESD, while that variable had a negative coordinate in Figure 6A.

## Discussion

### Temporal trajectories

The PTA that focused on the spatial structure of temporal dynamics yielded a temporal typology of wood features that opposed years where climatic conditions allowed growth to years where growth was limited with subsequent alteration of wood characteristics. The PTA showed that the main temporal structure was the separation between the slow growth during years 1972, 1979, 1980, 1996, and 2006 from the rest of the dates (Figure 6B). Some years appeared particularly favorable, such as 1994 and 2000. This pattern is fairly common to all trees because individuals experienced similar climatic conditions due to the relatively small size of the survey area. In the Alps, larch is recurrently defoliated by the larch budmoth (*Zeiraphera diniana*) [50]. Tree defoliation strongly decreases radial growth and produces anomalies in ring density variables during the year of defoliation and/or the year immediately following [51]. A. Roques (personal communication) provided historical records of defoliation by the larch budmoth in the Briançon. These data indicated that defoliation occurred in 1971, 1979, 1996, 1997, and 2006 in an experimental site located 6 km from our study site, at 1800 m asl. There is very close agreement between the direct observations of *Zeiraphera* defoliation in the URZF experimental site and the outputs of the PTA: the 1971 defoliation strongly decreased 1972 annual ring width and latewood formation; the 1979 defoliation affected both 1979 and 1980 rings; the 1996–1997 defoliation affected the 1996 and 1997 rings; and the 2006 defoliation only affected the 2006 annual ring.

Equally interesting is the trend identified in the second axis of the PCA of the compromise (Figure 6B). Such an effect may be related to longer-term climate evolution (e.g., temperature increase, as suggested in this study) (Figure S3). This is consistent with other results indicating that the mean annual temperature at Briançon increased by about 1.5°C from 1967–2007, which may have modified the annual ring structure (Figure S3) [38]. In the present survey, temporal changes of wood quality primarily involved a decrease of earlywood density and an increase of latewood density as well as a decrease of ring homogeneity. This is consistent with the conclusions of a recent survey on Siberian larch (a species closely related to European larch), which showed that warming favors wider earlywood cell lumen (i.e., lower density earlywood), thicker latewood walls (i.e., higher density latewood), denser maximum latewood, and wider rings [52]. Both the *Zeiraphera* defoliation and the temperature trend affected the temporal dynamics of tree-ring characteristics in a similar way for all trees, and a limited number of individuals displayed departure from that model for some ring descriptors (Figures 7 and S4). This rather homogeneous response of trees to climate could differ in other situations, such as field trials, where various families of genetically selected trees are planted (see below).

It should be noted that some factors may markedly affect tree ring variables, such as cambial age, competition, or heartwood formation. Here, we did not consider these factors because we focused on illustrating the methodology and the use of PTA, for which raw data appeared to be the best option. In some situations however, it may necessary to adjust the annual ring time series according to the objective of the study. While no adjustment is necessary for a wood quality study, it is generally mandatory for climatic or ecological studies.

### Spatial variability at the plot scale

The results reported in this paper highlight both the spatial structure of a set of descriptors of wood characteristics of European larch trees and the temporal trajectories of each individual tree across a series of 41 consecutive years. The PTA allowed us to clearly identify the presence of a spatial structure of wood descriptors that was common to all years (i.e., the first PTA presented in the study). This long-term stand structure appeared spatially dependent at a short scale. These results suggest that trees were submitted to contrasting environmental conditions, which affected their growth and the quality of wood in a manner that is constant across years. Radial growth traits, such as ring width (RW) and latewood width (LW), are mostly affected by environmental factors, among which the most influential is competition among trees [53]. For the ring density traits, the portion of the genetic variation in the total variance is generally much higher [54].

Soil variability is also a good candidate to explain stand spatial structure, as it is strongly variable in space at very different scales [55] and has substantial impact on plants [56]. The slope of the plot may also constitute an important environmental source of functional heterogeneity, as it affects water flow and often corresponds to spatial gradients of soil texture and overall soil fertility. The present study lacks environmental data allowing to fully explore the environmental drivers of stand spatial variability, but one highlight of PTA is that the compromise can easily be coupled with additional tools, such as spatial statistics. Trees of interest could be identified and monitored, and complementary data could be gathered, thus enabling a better exploration of their relationships with abiotic or biotic environmental factors (i.e., neighboring trees could be incorporated into additional analyses to assess the competition and other micro-environmental effects).

### The potential of PTA in tree-ring dataset analyses

The PTA proved to be a precious tool by allowing the proper identification of spatial structures common to all dates and exploration of their potential links with environmental drivers (e.g., soil fertility or water status). An obvious highlight of the approach was that it also allowed a direct access to temporal trajectories common to all trees. In many different cases, this would be helpful for exploring correlations with indices of climate change as well as identifying which ring descriptors (i.e., wood characteristics) are affected. Multivariate exploration of ring characteristics has been relatively uncommon. The PTA offers a complete space-time framework using a relatively simple mathematical basis, since the central tool is PCA [23] and software resources are available [47]. In our survey, the PTA allowed a proper separation between high and low frequency signals corresponding to inter-annual variations (at least partly linked to forest pest/insect outbreaks in this study) and long-term trends, respectively, possibly related to climate change. Because the PTA allowed for extraction, quantification, and hierarchization of these superimposed sources of variation, it is relevant for climate change research by processing the growing number of databases documenting tree chronologies that are compiled and made available [57].

In common garden experiments with genetic entities, the environmental heterogeneity is largely dictated by the design of the experiment (e.g., blocks of trees of various genetic lineages). The PTA would be helpful to stress the common temporal evolution of all trees, and the intrastructure would discern those lineages that differ from the common model and for which ring characteristics differ. The PTA would thus contribute to identifying lineages with potentially interesting wood characteristics. Considering that the annual ring variables constitute basic wood properties directly linked to the value of wood products, PTA could also be used as an efficient tool to describe variation of overall trunk structure from a wood-quality standpoint.

Finally, PTA can be seen as a pipeline (i.e., a set of connected data-processing elements). The output of some elements, such as the compromise table, can be used in various complementary analyses not strictly related to the PTA. We employed this strategy with the correlogram analysis, but the compromise table may also be taken as a summary of the data and co-analyzed with external data tables [14], [23], [30], [33], [34], [58]. Examples may also include genetic data describing each tree or data conveying the between-tree competition within the neighboring environment.

## Supporting Information

Figure S1 **Changes of tree ring variables from 1967–2007.**
(PDF)

Figure S2 **Spatial analysis of the partial triadic analysis depicting the temporal evolution of spatial structures.** Moran’s *I* correlogram of the tree scores upon the first axis of the PCA of the compromise table. Black (open) symbols indicate significant (non-significant) values at *p* = 0.05. The spatial structure proved globally significant (p<0.05) when assessed by means of the Holm’s correction test for simultaneous testing.
(PDF)

Figure S3 **Average daily minimum and maximum temperatures in Briançon (44°53′N, 6°38′E) averaged over the growing season of the European larch (March–September) from 1967–2007. Data source: Météo-France.**
(PDF)

Figure S4 **Intrastructure analysis of the partial triadic analysis depicting the spatial structure of temporal dynamics.** Scatter plot showing the coordinates of the ring variables projected onto the first axis of the principal component analysis of the compromise table for each tree.
(PDF)

Dataset S1 **Ring dataset.**
(TXT)
