# Supplementary material for: Dissecting the Space-Time Structure of Tree-Ring Datasets Using the Partial Triadic Analysis
Source: PLoS One. 2014 Sep 23;9(9):e108332. doi: 10.1371/journal.pone.0108332 (PMC4172773; doi:10.1371/journal.pone.0108332)

Supporting Information File Figure_S1.pdf

Figure S1: Changes of tree ring variables between 1967 and 2007. For each variable and each year, the measured values were averaged over the 149 studied trees. Measurements units are given in Table 1.

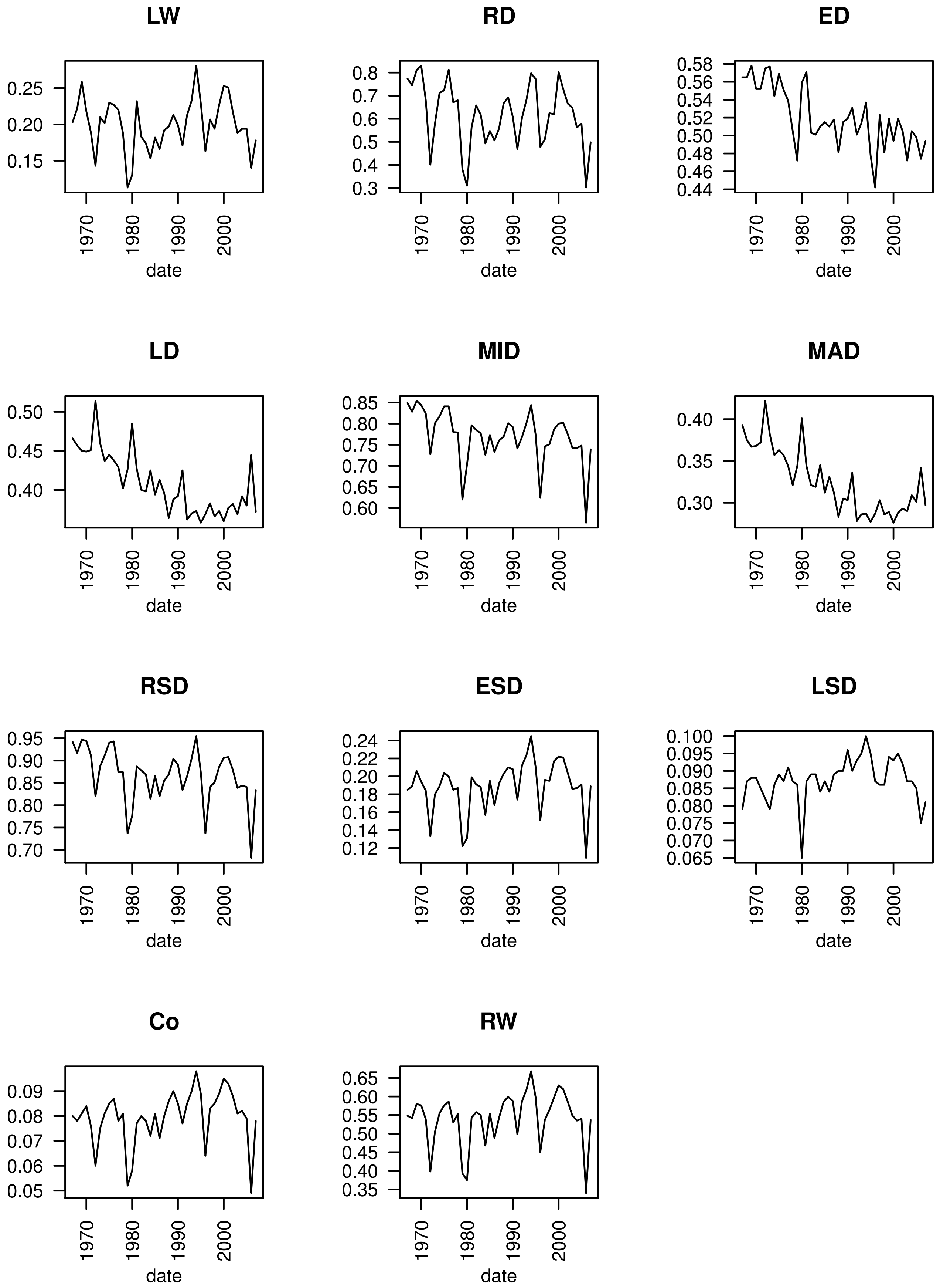

Supplement: Figure S1 — Changes of tree ring variables from 1967–2007. (PDF) [file pone.0108332.s001.pdf]
