# Supplementary material for: Dissecting the Space-Time Structure of Tree-Ring Datasets Using the Partial Triadic Analysis
Source: PLoS One. 2014 Sep 23;9(9):e108332. doi: 10.1371/journal.pone.0108332 (PMC4172773; doi:10.1371/journal.pone.0108332)

Supporting Information File Figure_S2.pdf

Figure S2: Spatial analysis of the PTA depicting the temporal evolution of spatial structures. Moran's I correlogram of the tree scores upon the first axis of the PCA of the compromise table. Black (open) symbols indicate significant (non significant) values at $\alpha = 0.05$. The spatial structure proved globally significant ($p < 0.05$) when assessed by means of the Holm's correction test for simultaneous testing.

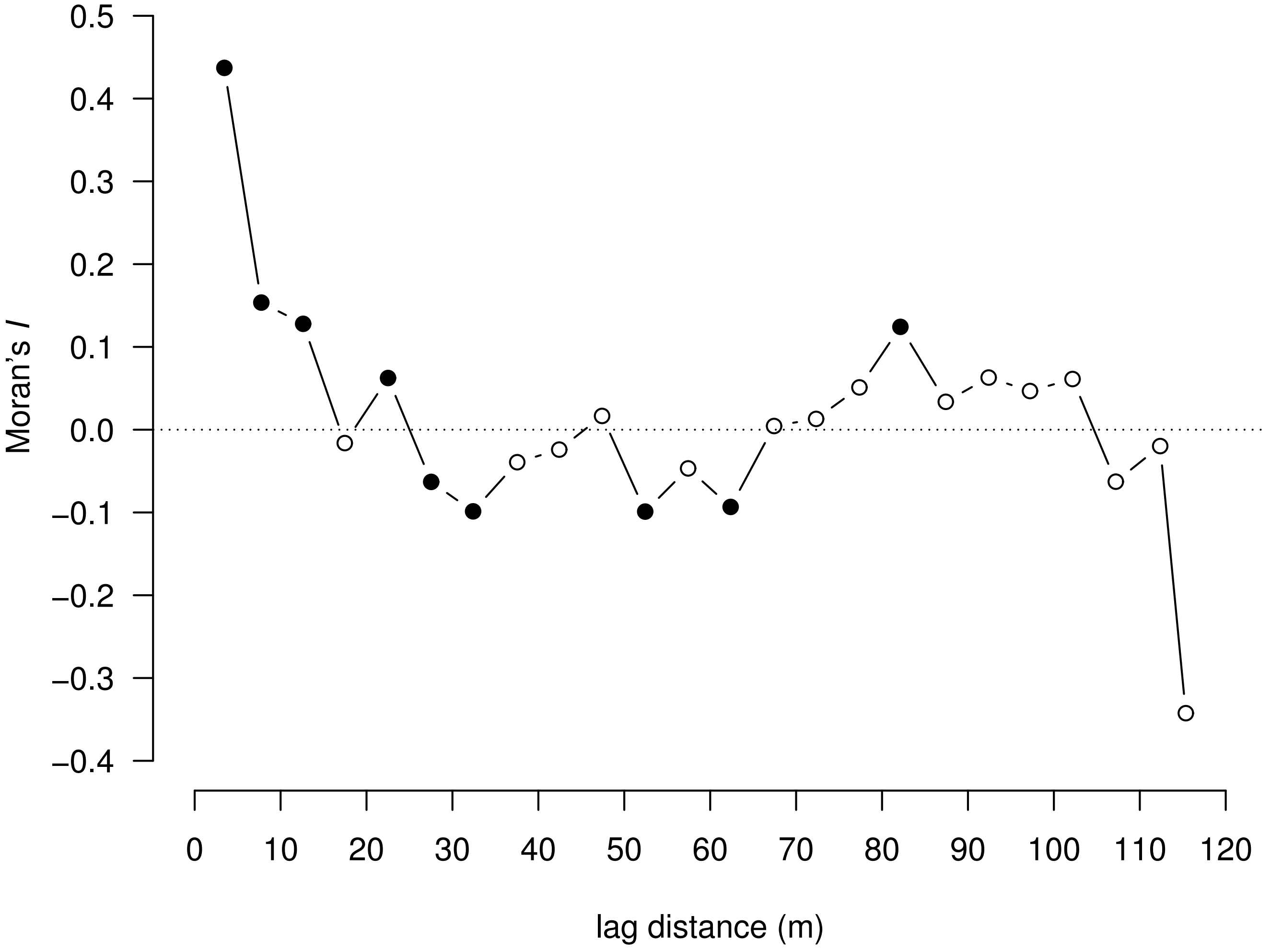

Supplement: Figure S2 — Spatial analysis of the partial triadic analysis depicting the temporal evolution of spatial structures. Moran’s I correlogram of the tree scores upon the first axis of the PCA of the compromise table. Black (open) symbols indicate significant (non-significant) values at p = 0.05. The spatial structure proved globally significant (p<0.05) when assessed by means of the Holm’s correction test for simultaneous testing. (PDF) [file pone.0108332.s002.pdf]
