# Supplementary material for: Dissecting the Space-Time Structure of Tree-Ring Datasets Using the Partial Triadic Analysis
Source: PLoS One. 2014 Sep 23;9(9):e108332. doi: 10.1371/journal.pone.0108332 (PMC4172773; doi:10.1371/journal.pone.0108332)

# Supporting Information File Figure_S3.pdf

**Figure S3**: Average daily minimum and maximum temperatures in Briançon (44°53′N 6°38′E) averaged over the growing season (March to September) between 1967 and 2007. Data source: Météo-France.

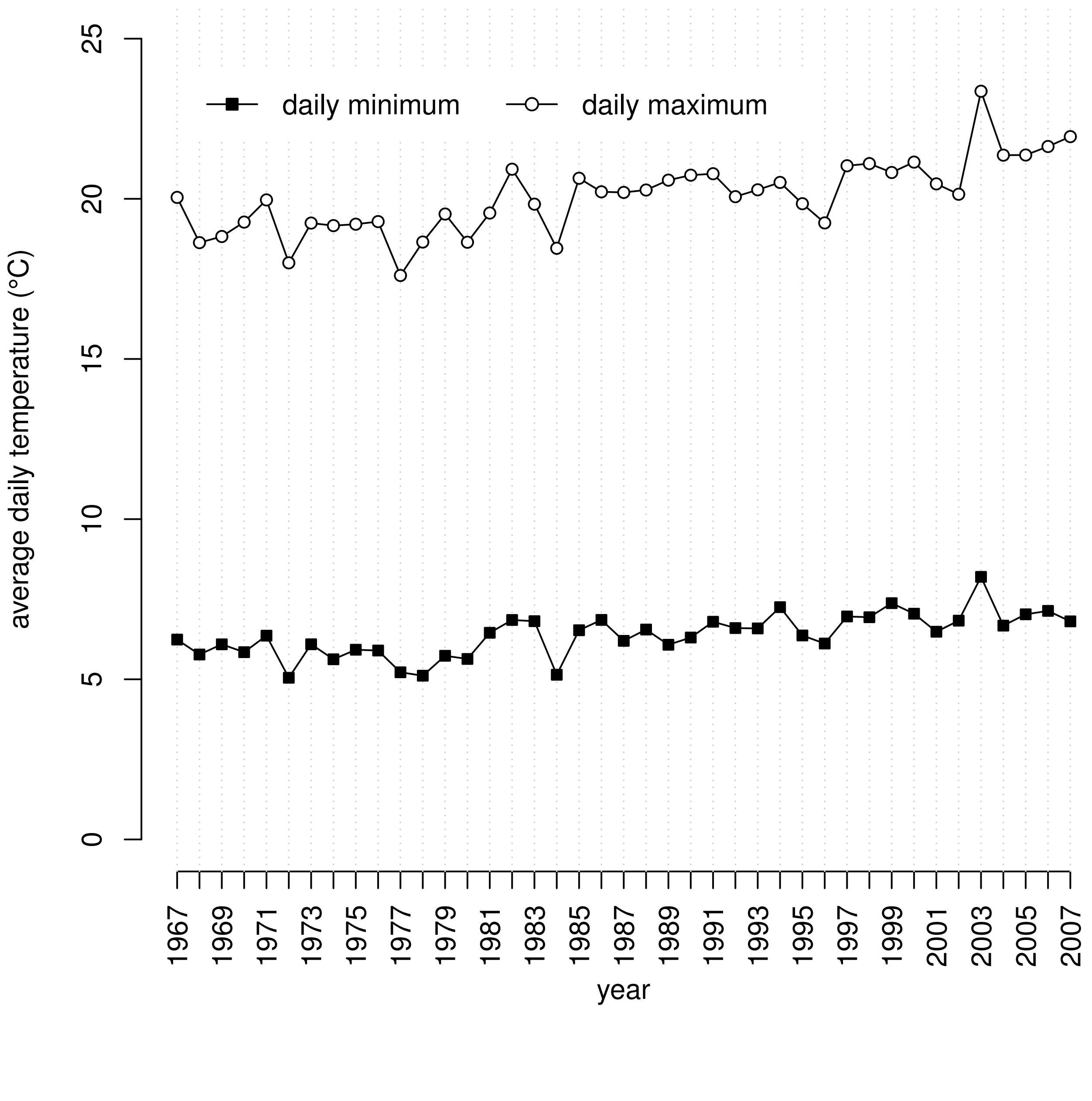

Supplement: Figure S3 — Average daily minimum and maximum temperatures in Briançon (44°53′N, 6°38′E) averaged over the growing season of the European larch (March–September) from 1967–2007. Data source: Météo-France. (PDF) [file pone.0108332.s003.pdf]
