# Supplementary material for: Dissecting the Space-Time Structure of Tree-Ring Datasets Using the Partial Triadic Analysis
Source: PLoS One. 2014 Sep 23;9(9):e108332. doi: 10.1371/journal.pone.0108332 (PMC4172773; doi:10.1371/journal.pone.0108332)

Supporting Information File Figure_S4.pdf

Figure S4: Intrastructure analysis of the PTA depicting the spatial structure of temporal dynamics. Scatter plot showing the coordinates of the ring variables projected onto the first axis of the PCA of the compromise table for each tree.

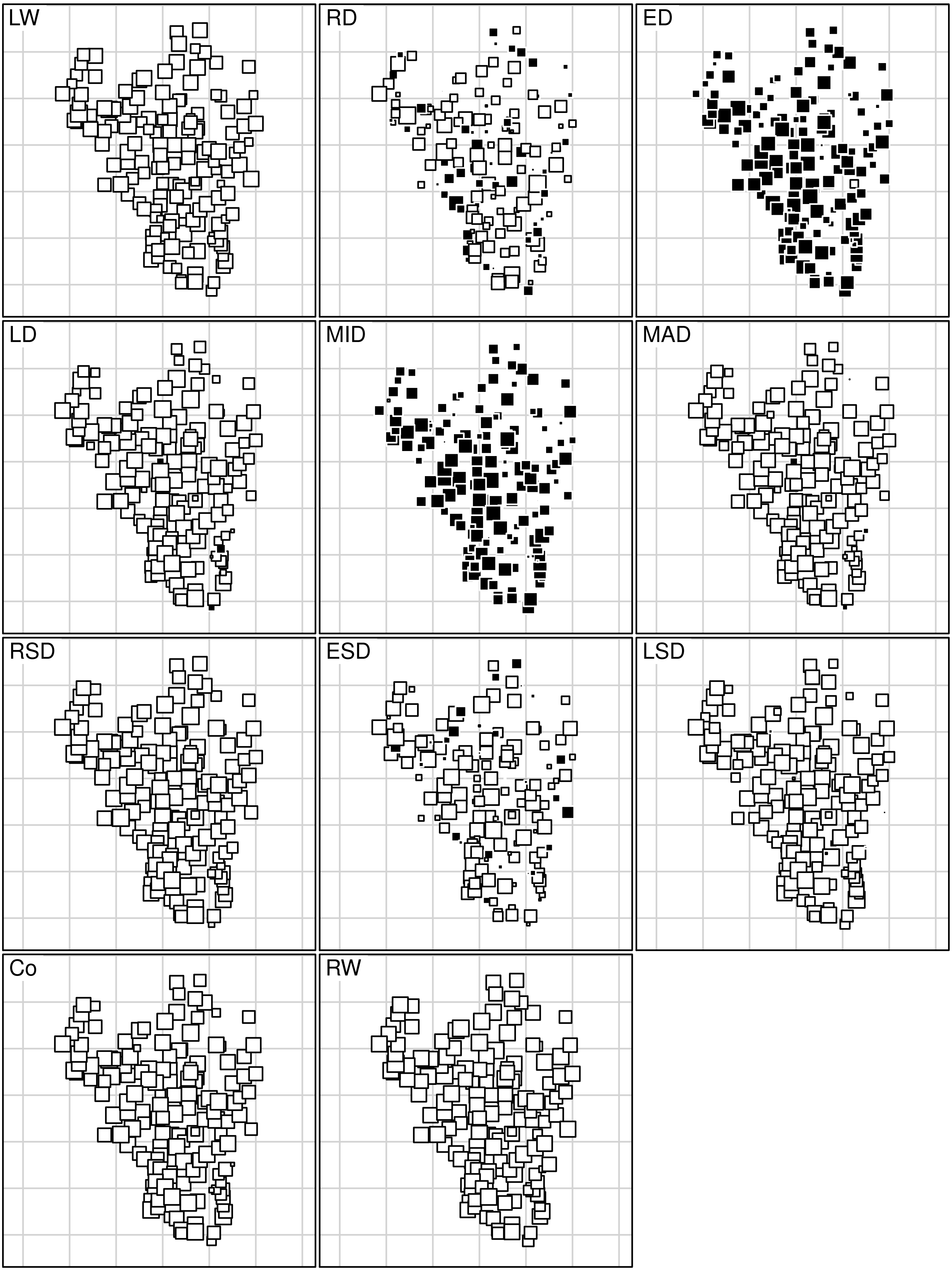

Supplement: Figure S4 — Intrastructure analysis of the partial triadic analysis depicting the spatial structure of temporal dynamics. Scatter plot showing the coordinates of the ring variables projected onto the first axis of the principal component analysis of the compromise table for each tree. (PDF) [file pone.0108332.s004.pdf]
